# Molecular characterization of *Mycobacterium bovis *strains isolated from cattle slaughtered at two abattoirs in Algeria

**DOI:** 10.1186/1746-6148-5-4

**Published:** 2009-01-27

**Authors:** Naima Sahraoui, Borna Müller, Djamel Guetarni, Fadéla Boulahbal, Djamel Yala, Rachid Ouzrout, Stefan Berg, Noel H Smith, Jakob Zinsstag

**Affiliations:** 1 Département Vétérinaire, Université Saad Dahlab, Blida, Algeria; 2 Centre Universitaire d'El-Tarf, El-Tarf, Algeria; 3 Department of Public Health and Epidemiology, Swiss Tropical Institute, Basel, Switzerland; 4 Département de Biologie, Université Saad Dahlab, Blida, Algeria; 5 Service de Tuberculose et des Mycobactéries, Institut Pasteur d'Algérie, Algiers, Algeria; 6 TB Research Group, Veterinary Laboratories Agency, Weybridge, UK; 7 Centre for the Study of Evolution, University of Sussex, Falmer, East Sussex, UK

## Abstract

**Background:**

Bovine Tuberculosis is prevalent in Algeria despite governmental attempts to control the disease. The objective of this study was to conduct, for the first time, molecular characterization of a population sample of *Mycobacterium bovis *strains isolated from slaughter cattle in Algeria. Between August and November 2007, 7250 animals were consecutively screened at the abattoirs of Algiers and Blida. In 260 animals, gross visible granulomatous lesions were detected and put into culture. Bacterial isolates were subsequently analysed by molecular methods.

**Results:**

Altogether, 101 bacterial strains from 100 animals were subjected to molecular characterization. *M. bovis *was isolated from 88 animals. Other bacteria isolated included one strain of *M. caprae*, four *Rhodococcus equi *strains, three Non-tuberculous Mycobacteria (NTM) and five strains of other bacterial species. The *M. bovis *strains isolated showed 22 different spoligotype patterns; four of them had not been previously reported. The majority of *M. bovis *strains (89%) showed spoligotype patterns that were previously observed in strains from European cattle. Variable Number of Tandem Repeat (VNTR) typing supported a link between *M. bovis *strains from Algeria and France. One spoligotype pattern has also been shown to be frequent in *M. bovis *strains from Mali although the VNTR pattern of the Algerian strains differed from the Malian strains.

**Conclusion:**

*M. bovis *infections account for a high amount of granulomatous lesions detected in Algerian slaughter cattle during standard meat inspection at Algiers and Blida abattoir. Molecular typing results suggested a link between Algerian and European strains of *M. bovis*.

## Background

*Mycobacterium bovis *is the causative agent of Bovine Tuberculosis (BTB) and belongs to the *Mycobacterium tuberculosis *Complex (MTC), a group of closely related bacteria causing tuberculosis in various mammalian hosts [1].

BTB has a major economic impact on livestock productivity [2], can persist in wildlife and thus affect entire ecosystems [3] and it is of public health concern due to its zoonotic potential [4-6]. Although still prevalent in the developed world [7-9], BTB today mostly affects developing countries, which lack the financial and human resources to control the disease [4,5]. BTB is also known to be prevalent in Algeria despite governmental attempts to control the disease [4,5]. However, control is restricted to abattoir meat inspection and biannual intra-dermal tuberculin skin testing of cattle from intensive dairy farms [10]. Moreover, the majority of Algerian cattle are not registered and cattle movement control schemes are not well established. Most of the BTB cases in Algeria are discovered during meat inspection in slaughter cattle at abattoirs when gross visible lesions typical of the disease are detected. However, in two recent studies in Chad and Uganda, Non-tuberculous Mycobacteria (NTM) were isolated from more than 40% of the animals exhibiting lesions [11,12]. This suggests that NTM infections in cattle might be of considerable importance in some African countries.

Spacer oligonucleotide typing (spoligotyping) and Variable Number of Tandem Repeat (VNTR) typing have been shown to be valuable tools for molecular epidemiology of *M. bovis *infections in a number of settings [7,13-15]. Spoligotyping can be used to identify distinct groups of strains, which can often be further differentiated by VNTR typing due to the latter's higher discriminatory power [1]. Extensive worldwide databases of spoligo- and VNTR typing patterns facilitate the comparison of results from different countries and help to elucidate the distribution and spread of strains (, [16]). The objectives of this study were to molecularly characterize a population sample of strains of *M. bovis *from Algeria using spoligotyping and VNTR typing and to identify potential exchange of strains with other regions.

## Results

At the abattoirs of Algiers and Blida in Algeria a consecutive case series of altogether 7250 slaughter animals was examined during standard meat inspection from August to November 2007. Lesions suggestive of BTB were sampled from 260 animals (apparent lesion prevalence: 3.6%; CI: 3.2 – 4.0%) and put into culture. Cultures from 106 animals with lesions did not show bacterial growth. Cultures from 20 animals showed contaminations and the remaining cultures from altogether 134 animals showed bacterial growth without visible contaminations. A sample of bacterial cultures from altogether 100 of these animals were further characterised by deletion-, spoligo- and VNTR typing, and sequencing of the 16S rRNA gene. Strains of *M. bovis *were identified by the deletion of RD4 in the genome sequence and by spoligotyping; *M. bovis *was detected in samples from 88 animals (table 1). In cultures of one of these animals in addition to *M. bovis*, a strain of *Rhodococcus equi *could be detected. One strain of the *M. caprae *clade was identified by the presence of the RD4 region and the deletion of RD12 in its genome sequence [1]. Altogether, four animals were shown to be infected with *R. equi *(including the animal with the *M. bovis/R. equi *mixed infection). NTM infections were detected in three animals (table 1). The 16S rRNA gene sequence of one NTM strain was most similar to the 16S rRNA gene sequence of *M. chitae *(97.7% sequence identity); the second NTM strain was most closely related to *M. brasiliensis *(98.4% sequence identity) and the third NTM strain showed highest sequence similarities to *M. acapulcensis *and *M. flavescens *(99.7% sequence identity for both). However, species identification of the NTM strains by 16S rRNA sequencing did not meet the requirements reported by Bosshard et al. and may have to be considered with caution [17]. *Ureibacillus thermosphaericus *and bacteria of the genus *Corynebacterium*, *Paenibacillus*, *Pseudomonas *and *Streptococcus *were each detected in samples from one animal (table 1).

**Table 1:** Bacteria isolated from tuberculous lesions of slaughtered cattle in Algeria

	**N**	**%**
**Animals screened**	**7250**	**100%**
**Animals with lesions**	**260**	**4%**
**Bacterial strains isolated:**	**101**	**100%**
Mycobacterium bovis*	88	87%
*Mycobacterium caprae*	1	1%
Non-tuberculous Mycobacteria	3	3%
Rhodococcus equi*	4	4%
*Ureibacillus thermosphaericus*	1	1%
*Corynebacterium *spp.	1	1%
*Paenibacillus *spp.	1	1%
*Pseudomonas *spp.	1	1%
*Streptococcus *spp.	1	1%

* One mixed infection of *M. bovis *and *R. equi *was detected in one animal

**Figure 1:**
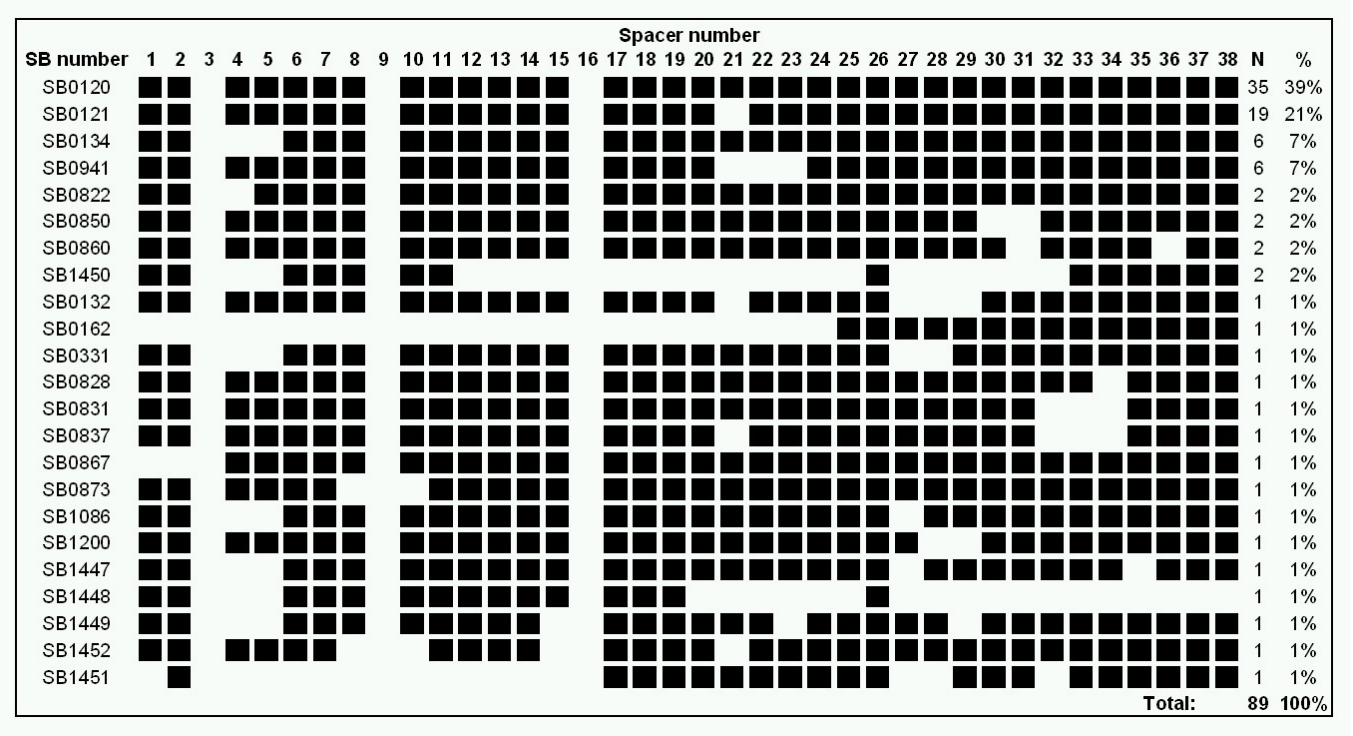
**Spoligotype patterns of MTC strains isolated from slaughter cattle at the abattoir of Algiers and Blida in Algeria**. Spacers 39–43 were absent from all spoligotype patterns. SB numbers were taken from . Previously unreported *M. bovis *spoligotype patterns were named SB1447, SB1448, SB1449 and SB1450 by  and the new *M. caprae *spoligotype pattern was named SB1451.

Spoligotyping of the 89 MTC strains isolated revealed altogether 23 different spoligotype patterns (figure 1); 18 of them had been previously reported and 5 were new, including the pattern of the *M. caprae *strain [18-20]. Previously unreported *M. bovis *spoligotype patterns were named SB1447, SB1448, SB1449 and SB1450 by  and the new *M. caprae *spoligotype pattern was named SB1451 (figure 1). The four most frequent spoligotype patterns (SB0120, SB0121, SB0134, SB0941) accounted for 40%, 22%, 7% and 7% of the *M. bovis *strains, respectively. Of the 22 *M. bovis *spoligotypes, 8 were clustered and the remaining 14 were unique patterns (allelic diversity = 0.78).

All the MTC strains were VNTR typed using the loci ETR A-E (see Additional file 1) [14]. For the *M. bovis *strains, 35 VNTR types with 16 clustered and 19 unique patterns could be identified (allelic diversity = 0.93). Spoligotyping combined with VNTR typing allowed us to distinguish 51 distinct types of *M. bovis *strains with 11 of them being clustered (allelic diversity = 0.95). The VNTR patterns of the four most common spoligotypes (SB0120, SB0121, SB0134 and SB0941) are shown in table 2. Spoligo- and VNTR types of all the 89 MTC strains are summarised in Additional file 1. It is noteworthy that the frequency of the various *M. bovis *genotypes detected did not markedly differ between the two study locations Algiers and Blida.

**Table 2:** VNTR allele profiles of strains with spoligotype patterns SB0120, SB0121, SB0134 and SB0941

**SB0120**		**SB0121**		**SB0134**		**SB0941**	
**VNTR type**	**Frequency**	**VNTR type**	**Frequency**	**VNTR type**	**Frequency**	**VNTR type**	**Frequency**
			
4 5 5 4* 3	6	6 4 3 4* 3	16	7 4 5 4* 3	3	6 5 3 4* 3	5
5 5 2 4* 3	4	4 3 5 3* 3	1	3 5 5 5* 3	1	6 7 3 4* 3	1
4 5 3 4* 3	3	5 3 5 4* 3	1	4 4 5 3* 4	1		
4 5 5 3* 3	3	6 4 4 4* 3	1	7 5 3 5* 3	1		
4 4 5 4* 3	2						
4 5 5 2* 3	2						
5 3 5 4* 3	2						
5 5 5 4* 4	2						
3 2 5 4* 3	1						
3 4 5 3* 3	1						
3 5 5 4* 4	1						
4 5 2 4* 3	1						
4 7 3 4* 3	1						
4 8 5 4* 3	1						
5 4 4 4* 3	1						
5 4 5 4* 3	1						
5 5 5 4* 3	1						
5 6 5 4* 3	1						
7 5 4 3* 3	1						
Total	35		19		6		6

VNTR allele profiles are indicated in order of loci ETR A-E

## Discussion

To our knowledge, this is the first study conducting the molecular characterization of strains of *M. bovis *isolated from slaughter cattle in Algeria. However, routine abattoir meat inspection and periodic intra-dermal tuberculin skin testing of cattle from intensive dairy farms have already previously revealed the presence of BTB in Algeria [4,5]. In our study, MTC infections were detected in 89/7250 animals (1% of all sampled animals). Considering the imperfect sensitivity of meat inspection and culture and the fact that only 100 of the 134 positive cultures have been characterized, the true prevalence of MTC infections in the sampled cattle population in fact may has been considerably higher. Ayele et al. previously reported a low sporadic incidence of BTB in Algeria [21]; therefore, our results indicate a more frequent occurrence of *M. bovis *infections in Algerian cattle than previously suspected.

Unlike reported for Chad and Uganda, where NTM strains were isolated from more than 40% of the animals with lesions [11,12], only 3 of 100 Algerian cattle showed NTM infections. *R. equi*, which was detected in samples of 4 animals, has so far not been reported in connection with cattle infections in Africa. Taken together, these results suggest that there may be a difference in the bacterial species causing pulmonary infection and lesion formation in cattle between Algeria and some countries of sub-Saharan Africa.

The calculated allelic diversity of spoligotype patterns among the *M. bovis *strains isolated in Algeria (0.78) was relatively low compared to France (0.91) but similar to strain diversities reported from the UK (0.79) and many other places in the world [1,7,13,22]. However, considering the fact that the sample of *M. bovis *genotypes characterized in our study may not be representative of the country wide population of *M. bovis*, the calculated allelic diversity should be considered with care.

Of the 22 *M. bovis *spoligotype patterns detected, 13 patterns, accounting for 89% of all the strains isolated, have previously been detected in strains isolated from French cattle [7]. The three spoligotype patterns most frequently detected in strains from Algeria (SB0120, SB0121, SB0134) are also the three most frequent types observed in France [7] and are also known to be common in strains from other continental European countries [8,23-25]. Live animal importation from Europe to Algeria is documented. It presumably started during the French colonial period (1830–1962), has continued up to the present time and has mainly aimed at increasing the Algerian population of highly productive dairy cattle. Therefore, the observed similarity of spoligotypes may reflect the introduction of *M. bovis *from mainland Europe to Algeria.

Interestingly, in a previous study we have identified strains of spoligotype pattern SB0134 (figure 1) as highly prevalent in cattle from neighbouring Mali [26]. Strains of that spoligotype pattern are also the third most frequent strains detected in France. VNTR typing results for loci ETR A-E were available for strains from Algeria and Mali but no matches were found (table 3). ETR A-D patterns for strains with spoligotype pattern SB0134 from the Normandy region in France have previously been published [27]. Pattern 7 4 5 4 for loci ETR A-D was detected in strains from France and Algeria and pattern 6 5 5 4 for loci ETR A-D was present in France and Mali (table 3). We obtained VNTR patterns of *M. bovis *strains isolated in France with spoligotype pattern SB0120 (M. Boschiroli, unpublished data) and found matching ETR A-D patterns for 10/19 Algerian *M. bovis *strains with spoligotype pattern SB0120 (data not shown).

**Table 3:** ETR typing results of strains with spoligotype pattern SB0134 from Algeria, Mali and the region of Normandy, France

**Algeria**	**Mali**	**France**
3 5 5 5* 3	5 5 5 4* 3	2 4 5 4
4 4 5 3* 4	6 5 5 4* 3^£^	3 3 3 4
7 4 5 4* 3^§^	7 5 5 4* 3	3 4 5 7
7 5 3 5* 3		4 5 5 4
		6 5 5 4^£^
		7 4 5 4^§^

Results from Mali and France have previously been published [26,27]. ^§ ^Matching patterns of strains from Algeria and France. ^£ ^Matching patterns of strains from Mali and France. The presence of a 24 bp deletion in one of the repeats of ETR D (in our analysis marked with *) was not declared in the French study.

Due to the small sample size in the studies in Mali and Algeria and the sampling at only two abattoirs in northern Algeria and one abattoir in Mali, we cannot infer the absence of strain exchange between Algeria and neighbouring Mali. However, our data indicates that some strains of *M. bovis *found in Algeria and Mali may have been independently introduced from France (or more generally continental Europe). Live animal importation from Europe into Algeria is continuing up to date. However, nowadays, a negative tuberculin skin testing result must be certified before importation. During the colonial period, import restrictions may have been less rigorous for cattle imported from France. Also, the increased efforts to control BTB in Europe during the second half of the 20^th ^century have lead to a decreased prevalence of the disease in cattle of many European countries [28]. Therefore, supposedly, introduction of strains of *M. bovis *into Algeria could have most likely occurred during the colonial time. However, occasional importation of diseased but undetected live cattle might still be possible. Introduction of *M. bovis *from Europe into other countries has been suggested several times [7,13,29-31] and further investigations on the relationship between European strains and strains from other parts of the world might be worthwhile in order to elucidate the global spread of *M. bovis*.

Due to the small sample size, the limited survey period and the sampling at only two abattoirs in northern Algeria, the population sample of *M. bovis *strains collected may not reflect the country-wide bacterial population structure. Therefore, frequencies of strains with a specific spoligotype do not necessarily mirror the actual frequency of these strains in the population. Moreover, it is possible that animals from some regions have been overrepresented in our sample. The origin of the animals could not be traced due to poor documentation and multiple selling-on of the animals prior to slaughter. However, we assume that the majority of animals were dairy cattle and that only few animals originated from the same herds. Due to the characteristics of the local livestock production system we further assume that the animals originated from a large area of northern Algeria with a majority of them presumably coming from the central northern part. Indeed there was evidence that some of the cattle slaughtered at the abattoir of Algiers originated from an approximately 300 km distant region around Sétif. The implementation of a tracing system for animals would be of great value to further enhance BTB surveillance through abattoir meat inspection or molecular epidemiological studies. A tracing system could possibly also help in the early detection of BTB outbreaks and their localization.

## Conclusion

This study presents the first molecular characterisation of a population sample of strains of *M. bovis *isolated from Algerian cattle. BTB accounted for a high amount of granulomatous lesions detected in Algerian slaughter cattle during standard meat inspection at Algiers and Blida abattoir. Spoligotyping as well as VNTR typing results suggest a close link between the strains isolated from Algerian cattle and *M. bovis *strains from mainland Europe. This study highlights the importance of both spoligotype and VNTR typing databases and standardised protocols to assist global molecular epidemiological investigations of *M. bovis *infections.

## Methods

### Sample collection

Samples were collected between August and November 2007 from a sequential series of slaughter cattle at two abattoirs in Algeria (in Algiers and Blida), approximately 50 km apart from each other. The cattle population consisted mainly of young males and old cows with males being significantly more often slaughtered at the abattoir of Algiers and cows more often slaughtered at the abattoir of Blida. Of altogether 7250 animals examined, 93% of the cattle were exotic breeds (Holstein and Montbelliard), 6% were cross-breeds and only 1% local breeds. Altogether, 4980 animals (69%) were males and 2270 (31%) were females. The origin of the cattle could not be traced due to poor documentation. We assume that the majority of animals were dairy cattle and only few animals from the same herds. The animals encountered at Algiers and Blida abattoir possibly originated from a large area of northern Algeria with a majority of them presumably coming from the central northern part. Tissue samples of 260 animals with gross visible lesions were collected. The samples were transported on ice to the Institut Pasteur d'Alger for further processing.

### Tissue preparation, culture and DNA extraction

At Institut Pasteur, Algiers, specimens from all 260 animals, which exhibited gross visible lesions, were dissected and manually homogenised using a mortar. Samples were decontaminated by addition of 4 ml of 4% H_2_SO_4 _and neutralised with 6% NaOH using bromothymol blue as an indicator for the pH. Two Löwenstein-Jensen slants, supplemented with either sodium pyruvate or glycerol, were inoculated with 3 ml of the suspension and incubated at 37°C until bacterial growth was visible or for at least 12 weeks. Presence of Acid-Fast Bacilli was tested by Ziehl-Neelsen staining and microscopy. Cultures from 106 animals did not show bacterial growth. Cultures from 20 animals showed contaminations and the remaining cultures from altogether 134 animals showed bacterial growth without visible contaminations.

A sub-sample of 101 bacterial cultures from 100 animals was sent to the National Reference Centre for Mycobacteria in Zurich, Switzerland. DNA of all cultures was extracted using the InstGene™ Matrix (Bio-Rad^®^).

### Molecular characterisation

Spoligotyping was conducted at the Veterinary Laboratories Agency in Weybridge, UK as previously described [32]. VNTR typing was carried out as previously described using primers targeting the loci ETR A, ETR B, ETR C, ETR D and ETR E according to protocols of Frothingham et al. [14]. The ETR-D locus contains a 24 bp deletion in one of the repeats and the naming convention indicates the presence of this deletion by a * i.e. 4* (= 3 × 77 bp repeats and one 53 bp repeat) [33]. Allelic diversity was calculated according to the method of Selander et al. [34]. Strains were identified as *M. bovis *by the absence of the region RD4 and as *M. caprae *by the absence of RD12 and presence of RD4 as described by Brosch et al. [35]. The 16S rRNA gene amplification and sequencing was carried out as described by Zucol et al. [36]. Species identification was carried out by comparison with the sequences of the SmartGene Integrated Database Network System (IDNS™) 3.4.0. Criteria for species identification were taken from Bosshard et al. [17].

## Authors' contributions

NS conception and design of the study, sampling of animals, culturing of Mycobacteria, VNTR typing. BM principal supervision of molecular typing, and molecular analysis of bacterial strains, writing of the manuscript. DG supervision of the project and main supervision of NS. FB supervision of laboratory work and cultures at Institut Pasteur, Algiers. DY supervision of laboratory work and cultures at Institut Pasteur, Algiers. RO fieldwork and sample collection. SB contribution in paper writing and support for molecular typing NHS contribution in analysis of the data, important intellectual contribution for population genetical analysis. JZ supervision of the project, acquisition of parts of the funds, important intellectual contributions. All authors read and approved the final manuscript.

## Supplementary Material

Additional file 1 **Complete molecular typing data of MTC strains isolated from Algerian cattle**. The data provided shows the original sample name, spoligotype number according to  and ETR A-E typing results for all 89 MTC strains isolated from cattle carcasses at Algiers and Blida abattoir in Algeria. Click here for file

## References

[B1] Smith NH, Gordon SV, de la Rua-Domenech R, Clifton-Hadley RS, Hewinson RG (2006). Bottlenecks and broomsticks: the molecular evolution of Mycobacterium bovis. Nature Reviews Microbiology.

[B2] Zinsstag J, Schelling E, Roth F, Kazwala RR, Thoen CO, Steele JH, Gilsdorf MJ (2006). Economics of bovine tuberculosis. Mycobacterium bovis Infection in Animals and Humans.

[B3] Renwick AR, White PC, Bengis RG (2006). Bovine tuberculosis in southern African wildlife: a multi-species host-pathogen system. Epidemiol Infect.

[B4] Cosivi O, Grange JM, Daborn CJ, Raviglione MC, Fujikura T, Cousins D (1998). Zoonotic tuberculosis due to Mycobacterium bovis in developing countries. Emerg Infect Dis.

[B5] Ayele WY, Neill SD, Zinsstag J, Weiss MG, Pavlik I (2004). Bovine tuberculosis: an old disease but a new threat to Africa. Int J Tuberc Lung Dis.

[B6] Thoen C, Lobue P, de KI (2006). The importance of Mycobacterium bovis as a zoonosis. Vet Microbiol.

[B7] Haddad N, Ostyn A, Karoui C, Masselot M, Thorel MF, Hughes SL (2001). Spoligotype diversity of Mycobacterium bovis strains isolated in France from 1979 to 2000. Journal of Clinical Microbiology.

[B8] Aranaz A, Liebana E, Mateos A, Dominguez L, Vidal D, Domingo M (1996). Spacer oligonucleotide typing of Mycobacterium bovis strains from cattle and other animals: A tool for studying epidemiology of tuberculosis. Journal of Clinical Microbiology.

[B9] Smith NH, Dale J, Inwald J, Palmer S, Gordon SV, Hewinson RG (2003). The population structure of Mycobacterium bovis in Great Britain: clonal expansion. Proc Natl Acad Sci USA.

[B10] (2007). Ministère de l'Agriculture et du développement Rural de l'Algérie. Bulletin Sanitaire Vétérinaire.

[B11] Oloya J, Kazwala R, Lund A, Opuda-Asibo J, Demelash B, Skjerve E (2007). Characterisation of mycobacteria isolated from slaughter cattle in pastoral regions of Uganda. BMC Microbiol.

[B12] Diguimbaye-Djaïbe C, Vincent V, Schelling E, Hilty M, Ngandolo R, Mahamat HH (2006). Species identification of non-tuberculous mycobacteria from humans and cattle of Chad. Schweiz Arch Tierheilkd.

[B13] Njanpop-Lafourcade BM, Inwald J, Ostyn A, Durand B, Hughes S, Thorel MF (2001). Molecular typing of Mycobacterium bovis isolates from Cameroon. J Clin Microbiol.

[B14] Frothingham R, Meeker-O'Connell WA (1998). Genetic diversity in the Mycobacterium tuberculosis complex based on variable numbers of tandem DNA repeats. Microbiology.

[B15] Hilty M, Diguimbaye C, Schelling E, Baggi F, Tanner M, Zinsstag J (2005). Evaluation of the discriminatory power of variable number tandem repeat (VNTR) typing of Mycobacterium bovis strains. Vet Microbiol.

[B16] Brudey K, Driscoll JR, Rigouts L, Prodinger WM, Gori A, Al-Hajoj SA (2006). Mycobacterium tuberculosis complex genetic diversity: mining the fourth international spoligotyping database (SpoIDB4) for classification, population genetics and epidemiology. Bmc Microbiology.

[B17] Bosshard PP, Abels S, Zbinden R, Bottger EC, Altwegg M (2003). Ribosomal DNA sequencing for identification of aerobic gram-positive rods in the clinical laboratory (an 18-month evaluation). Journal of Clinical Microbiology.

[B18] Brudey K, Driscoll JR, Rigouts L, Prodinger WM, Gori A, Al-Hajoj SA (2006). Mycobacterium tuberculosis complex genetic diversity: mining the fourth international spoligotyping database (SpolDB4) for classification, population genetics and epidemiology. BMC Microbiol.

[B19] Kubica T, Rusch-Gerdes S, Niemann S (2003). Mycobacterium bovis subsp. caprae caused one-third of human M. bovis-associated tuberculosis cases reported in Germany between 1999 and 2001. J Clin Microbiol.

[B20] Prodinger WM, Brandstatter A, Naumann L, Pacciarini M, Kubica T, Boschiroli ML (2005). Characterization of Mycobacterium caprae isolates from Europe by mycobacterial interspersed repetitive unit genotyping. J Clin Microbiol.

[B21] Boddinghaus B, Rogall T, Flohr T, Blocker H, Bottger EC (1990). Detection and identification of mycobacteria by amplification of rRNA. J Clin Microbiol.

[B22] Diguimbaye-Djaïbe C, Hilty M, Ngandolo R, Mahamat HH, Pfyffer GE, Baggi F (2006). Mycobacterium bovis isolates from tuberculous lesions in Chadian zebu carcasses. Emerg Infect Dis.

[B23] Pavlik I, Dvorska L, Bartos M, Parmova I, Melicharek I, Jesenska A (2002). Molecular epidemiology of bovine tuberculosis in the Czech Republic and Slovakia in the period 1965–2001 studied by spoligotyping. Veterinarni Medicina.

[B24] Allix C, Walravens K, Saegerman C, Godfroid J, Supply P, Fauville-Dufaux M (2006). Evaluation of the epidemiological relevance of variable-number tandem-repeat genotyping of Mycobacterium bovis and comparison of the method with IS6110 restriction fragment length polymorphism analysis and spoligotyping. J Clin Microbiol.

[B25] Serraino A, Marchetti G, Sanguinetti V, Rossi MC, Zanoni RC, Catozzi L (1999). Monitoring of transmission of tuberculosis between wild boars and cattle: Genotypical analysis of strains by molecular epidemiology techniques. Journal of Clinical Microbiology.

[B26] Müller B, Steiner B, Bonfoh B, Fane A, Smith NH, Zinsstag J (2008). Molecular characterisation of Mycobacterium bovis isolated from cattle slaughtered at the Bamako abattoir in Mali. BMC Vet Res.

[B27] Zanella G, Durand B, Hars J, Moutou F, Garin-Bastuji B, Duvauchelle A (2008). Mycobacterium bovis in wildlife in France. J Wildl Dis.

[B28] Caffrey JP (1994). Status of bovine tuberculosis eradication programmes in Europe. Vet Microbiol.

[B29] Cousins D, Williams S, Liebana E, Aranaz A, Bunschoten A, Van Embden J (1998). Evaluation of four DNA typing techniques in epidemiological investigations of bovine tuberculosis. Journal of Clinical Microbiology.

[B30] Costello E, O'Grady D, Flynn O, O'Brien R, Rogers M, Quigley F (1999). Study of restriction fragment length polymorphism analysis and spoligotyping for epidemiological investigation of Mycobacterium bovis infection. J Clin Microbiol.

[B31] Zumarraga MJ, Martin C, Samper S, Alito A, Latini O, Bigi F (1999). Usefulness of spoligotyping in molecular epidemiology of Mycobacterium bovis-related infections in South America. Journal of Clinical Microbiology.

[B32] Kamerbeek J, Schouls L, Kolk A, van Agterveld M, van Soolingen D, Kuijper S (1997). Simultaneous detection and strain differentiation of Mycobacterium tuberculosis for diagnosis and epidemiology. J Clin Microbiol.

[B33] Michel AL, Hlokwe TM, Coetzee ML, Mare L, Connoway L, Rutten VP (2008). High Mycobacterium bovis genetic diversity in a low prevalence setting. Vet Microbiol.

[B34] Selander RK, Caugant DA, Ochman H, Musser JM, Gilmour MN, Whittam TS (1986). Methods of multilocus enzyme electrophoresis for bacterial population genetics and systematics. Appl Environ Microbiol.

[B35] Brosch R, Gordon SV, Marmiesse M, Brodin P, Buchrieser C, Eiglmeier K (2002). A new evolutionary scenario for the Mycobacterium tuberculosis complex. Proc Natl Acad Sci USA.

[B36] Zucol F, Ammann RA, Berger C, Aebi C, Altwegg M, Niggli FK (2006). Real-time quantitative broad-range PCR assay for detection of the 16S rRNA gene followed by sequencing for species identification. J Clin Microbiol.

